# Population phenomena inflate genetic associations of complex social traits

**DOI:** 10.1126/sciadv.aay0328

**Published:** 2020-04-15

**Authors:** Tim T. Morris, Neil M. Davies, Gibran Hemani, George Davey Smith

**Affiliations:** 1 Medical Research Council Integrative Epidemiology Unit, University of Bristol, Bristol BS8 2BN, UK.; 2 Bristol Medical School, University of Bristol, Oakfield House, Oakfield Grove, Bristol BS8 2BN, UK.; 3 K.G. Jebsen Center for Genetic Epidemiology, Department of Public Health and Nursing, NTNU, Norwegian University of Science and Technology, Norway.

## Abstract

Heritability, genetic correlation, and genetic associations estimated from samples of unrelated individuals are often perceived as confirmation that genotype causes the phenotype(s). However, these estimates can arise from indirect mechanisms due to population phenomena including population stratification, dynastic effects, and assortative mating. We introduce these, describe how they can bias or inflate genotype-phenotype associations, and demonstrate methods that can be used to assess their presence. Using data on educational achievement and parental socioeconomic position as an exemplar, we demonstrate that both heritability and genetic correlation may be biased estimates of the causal contribution of genotype. These results highlight the limitations of genotype-phenotype estimates obtained from samples of unrelated individuals. Use of these methods in combination with family-based designs may offer researchers greater opportunities to explore the mechanisms driving genotype-phenotype associations and identify factors underlying bias in estimates.

## Teaser

In this paper, we review and demonstrate how population phenomena can inflate genotype-phenotype associations.

## INTRODUCTION

Genotype-phenotype associations inferred from genetic data can be used to provide insight into the genetic architecture of complex traits, interrogate causal and noncausal associations between different phenotypes, and create phenotypic predictors (*1*–*3*). In most situations, these applications depend upon a narrow definition of genotype-phenotype association—that there is a causal path from an individual’s genotype to the same individual’s phenotype. However, the common practice of using samples of unrelated individuals to estimate genotype-phenotype associations is liable to bias, where other causal paths can confound these associations. For example, population dynamic phenomena such as population stratification, dynastic effects, and assortative mating can induce correlations through confounding between genotypes and phenotypes. These processes do not reflect the causal pathways that are generally intended to be identified.

In this paper, we first review the strategies for estimating genotype-phenotype associations in samples of unrelated individuals and then describe in detail the population dynamic phenomena that could bias these estimates away from the causal parameter. We then demonstrate empirical examples of these with a focus on socioeconomic phenotypes, suggest tools for detecting these biases, and discuss some potential consequences of, and solutions to them.

### Estimation of genotype-phenotype associations

**Fig. 1:**
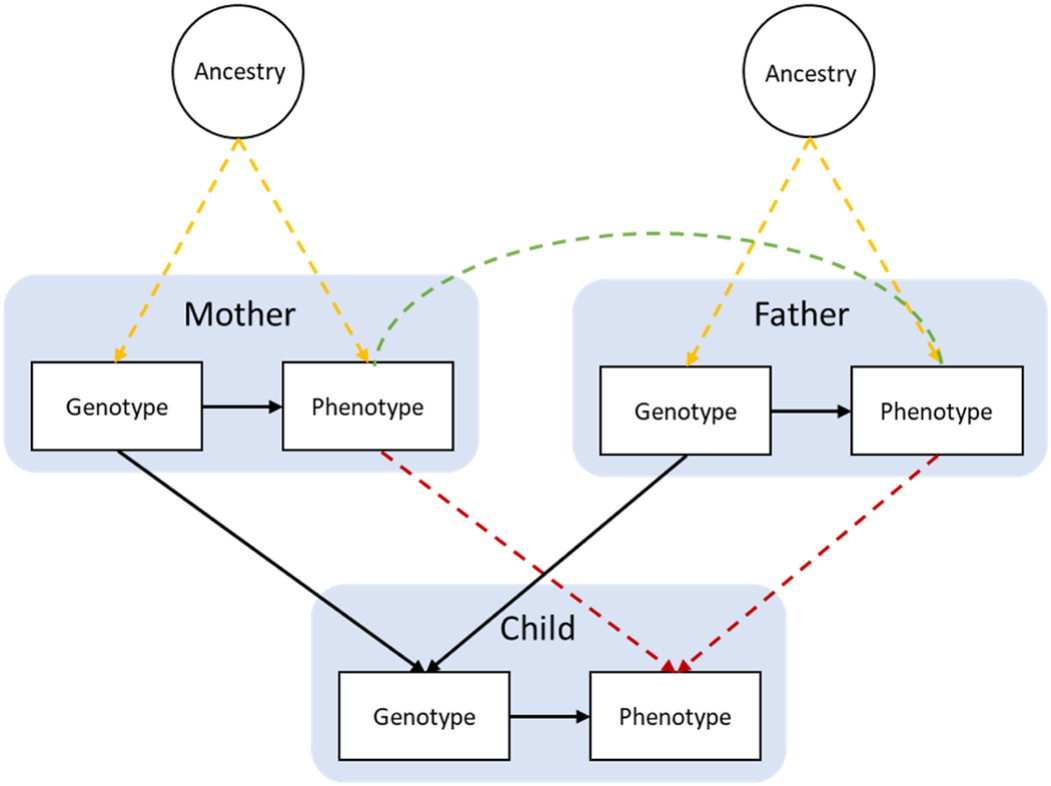
Causal models of structures underlying genetic associations. Population stratification due to ancestral differences (yellow lines), dynastic effects (red lines), and assortative mating (green line). Arrows represent direction of effect; nondirected lines represent simultaneous assortment. Note: Assortative mating by phenotype will lead to genotypic correlation (*62*).

Fisher (*4*) partitioned genotype-phenotype associations into two components, although the terms for these are not used consistently (*5*). For simplicity, we will refer to them as variant substitution effects and confounding effects. Variant substitution effects can be thought of as the (counterfactual) change in an individual’s phenotype that would occur as a result of changing that individual’s genotype from conception (holding all else constant). In most cases, this type of effect is the target of any genotype-phenotype association analysis. The mechanism that cascades from a variant substitution may be entirely molecular, for example, altering gene expression that leads to disease, or it may be more complex and external, for example, influencing behavior that leads to environmental changes that, in turn, influence the phenotype. In both cases, there is a causal path from an individual’s genotype to their phenotype that reflects a counterfactual model. If this path of interest from genotype to phenotype is confounded, genotype-phenotype associations will not (solely) reflect underlying causal mechanisms but will be biased. Various population phenomena such as population stratification (*6*), dynastic effects (*7*), and assortative mating (*8*, *9*) can introduce such confounding (Fig. 1 and Box 1) (*2*, *10*). These population phenomena can be considered to inflate the true values of population estimates and represent the inaccuracy of the hypothetical counterfactual of substituting a sampled individual’s genotype on their phenotype.

Box 1 **Structures that can induce associations between an individual’s genotype and their phenotype.**

**1. Variant substitution effects**

Variant substitution effects can be thought of as the (counterfactual) change in an individual’s phenotype that would occur as a result of changing that individual’s genotype from conception (holding all else constant). They can be estimated for a single phenotype (univariate genetic association) or pairs of phenotypes (bivariate genetic association, genetic correlation, or Mendelian randomization). In most cases, this type of effect is the target of any genotype-phenotype association analysis.

**2. Population stratification**

Population stratification refers to confounding introduced to associations between population structure and phenotype by systematic differences in allele frequencies across subpopulations. This arises from ancestry differences due to nonrandom mating and subsequent genetic drift of allele frequencies between subpopulation groups, historically caused by geographic and physical boundaries. If phenotypes also differ systematically between subpopulations, population stratification can lead to genotype-phenotype associations despite no causal relationship between the genotype and the phenotype.

**3. Dynastic effects**

Further to influencing offspring phenotype through genetic inheritance, parental genotype can indirectly influence offspring phenotype through its expression in the parental phenotype. Where this occurs, offspring may inherit both phenotype-associated SNPs and phenotype-associated environments from parents, leading to biased genetic associations (*7*). For example, SNPs positively associated with education in the parent’s generation may lead to the creation of educationally rich environments (such as an increase in books in the household), which will have a positive impact upon the child’s educational attainment. Here, a variant substitution effect in the parent is inducing confounding at the level of the individuals being studied. Dynastic effects refer to this “inheritance” of environment in addition to genotype.

**4. Assortative mating**

Assortative mating refers to the process by which spouses select each other based on certain phenotypic characteristics. If selected phenotypes have a genotypic component, then phenotypic selection induces greater genetic similarity between spouses than in the general population. The correlations that are induced between genotype and phenotype by phenotypic assortment will lead to biased estimates of the causal effect of genotype on phenotype in subsequent generations (*20*–*22*). While offspring inheritance of genotype is random conditional upon parent’s genotype, assortative mating induces nonrandom inheritance patterns across groups based on phenotype.

Genotype-phenotype associations are commonly estimated in three ways: single-nucleotide polymorphism (SNP) heritability, which represents the total genetic component of a trait estimated from variation in all measured SNPs; genetic correlation, which represents the correlation in effects of all measured SNPs on two or more phenotypes; and genetic associations, which represent how a phenotype is influenced by a specific SNP. SNP heritability and genetic correlation are estimated from whole genome data with methods such as genomic-relatedness-based restricted maximum likelihood (GREML) (*11*) and linkage disequilibrium (LD) score regression (*12*), while genetic associations are estimated as per-SNP effects in genome-wide association studies (GWASs) using linear or logistic regression. Throughout, we refer to these collectively as genotype-phenotype associations. We focus on evaluating how various population-level phenomena bias the parameters that can be estimated from whole genome-based approaches such as SNP heritability and genetic correlation, but the biases we describe can also inflate per-SNP estimates obtained from GWAS. We note that whole genome methods are additionally susceptible to a separate set of biases that have been under considerable scrutiny, which arise when the observed SNPs follow different distributions to the unknown causal variants (*13*), although they will not be discussed further here.

### The population-level phenomena that can contaminate genotype-phenotype associations

One mechanism that may bias genetic associations is population stratification (Fig. 1, yellow paths), where confounding of genotype-phenotype associations is driven by population structure (*14*). Population structure refers to systematic differences in allele frequencies between subpopulations (which often appears as geographical structure) due to ancestry (*6*). Because phenotypes are often geographically patterned, spurious genotype-phenotype associations (both heritability and genetic correlation) can arise even when a variant substitution effect on the phenotype does not exist. An oft-repeated example is that SNPs that have different frequencies in East Asian and European populations will be associated with chopstick use, although the reasons underlying chopstick use are cultural rather than genetic (*15*). Bias due to population stratification is commonly controlled for by restricting samples to a homogenous population and adjusting models for principal components of genotype, which capture common differences between subpopulations in allele frequencies. A recent study, however, demonstrated that geographical structure remains even after controlling for the first 100 principal components in large-scale biobanks, far in excess of the 10 or 20 components commonly controlled for (*16*). While it is not possible to prove that adjusting for principal components has controlled for all differences within the sample, one way to assess the impact of population stratification is to compare estimates obtained from unadjusted models and models that adjust for principal components. Attenuation in estimated effect sizes after principal component adjustment can provide evidence of population stratification, and the extent of this may be gauged by the extent of attenuation. However, in studies with a geographically homogenous sampling framework, this may be insufficient (*16*). Between-sibling study designs offer a robust solution because Mendel’s first and second laws of independent segregation and assortment ensure that genetic differences between siblings are not correlated with environment (*17*, *18*).

Genetic associations can also be biased by dynastic effects (Fig. 1, red paths), whereby inherited SNPs operate indirectly on offspring phenotype via their effects in the parents’ phenotype. For example, suppose that education-associated SNPs at the parental generation contribute to the creation of education-enriching environments through the provision of books in the household. It follows that children of more educated parents will be more likely to inherit both education-associated SNPs (the biological path from offspring genotype to offspring education) and education-associated environments (the nonbiological path from parental genotype to offspring education). This is a form of gene-environment correlation and can be thought of as a double contribution of genotype. Thus, social or environmental transmission effects can affect genotype-phenotype associations, leading to biased estimates of the causal effect of genotype on phenotype. It is important to note that under this model, the confounding effect is due to a variant substitution effect, although the variant substitution occurred not in the individual being analyzed but in their parents. It is possible that dynastic effects explain the relatively low estimates of the contribution of the shared environment from twin studies, which attribute these “genetic nurture” effects to the additive (heritable) effects of genetics. There is a large body of evidence suggesting that social phenotypes such as education and socioeconomic position (SEP) are socially transmitted across generations (*19*), and it is likely that genetic associations with these phenotypes will be affected by dynastic effects. The presence of dynastic effects can be tested and estimated with data on mother-father-offspring trios or siblings (*17*). Using polygenic scores, the raw association between offspring genotype and phenotype can be compared with its association when adjusted for maternal and paternal genotype. Attenuation of the raw association and direct (conditional) association between parental genotype and offspring phenotype supports an indirect effect of parental genotype on offspring phenotype and therefore the presence of dynastic effects. It is also possible to use nontransmitted parental SNPs to create a genetic nurture polygenic score (*7*). Because nontransmitted SNPs can only influence offspring phenotype indirectly, association between a nontransmitted score and offspring phenotype supports dynastic effects. Relatedness disequilibrium regression, which investigates changes in phenotypic similarity by relatedness among samples of siblings, can also be used to estimate bias in heritability estimates caused by environmental effects (*10*). These methods all require data on genotyped mother-father-offspring trios and will be facilitated by large family-based studies.

Assortative mating (Fig. 1, green path) may also induce genetic associations between phenotypes. Assortative mating refers to the nonrandom pairing of spouses across the population and arises from mate selection based on phenotypic characteristics and social homogamy. There is evidence for assortative mating on a range of phenotypes including education and SEP (*8*, *9*). Where phenotypes that are selected on have a genetic component, assortative mating will lead to spouses being more genetically similar to each other than to randomly selected individuals from a population. That is, phenotypic assortative mating across a population increases the likelihood of people mating with partners who are more genetically similar. While random mating would ensure even distribution of allele frequencies at the population level, assortative mating leads to systematic differences in allele frequencies (population stratification) and subsequent deviations from Hardy-Weinberg equilibrium that is reproduced over generations (*8*). Assortative mating will lead to a disproportionate enrichment or depletion of education-associated alleles within spouse couples and increased homozygosity, long-range linkage, and genetic variation in offspring across a population, biasing genotype-phenotype associations (*8*, *20*, *21*). For example, offspring of parents with higher education are more likely to have a greater number of education-increasing alleles than offspring of parents with lower education. If spouses sort on different traits (i.e., cross trait assortative mating), then assortment can also induce genetic correlations between traits in offspring (*22*). Assortative mating can lead to enhanced population stratification if it is subpopulation specific (*23*) and to disproportionate inheritance of the environment in addition to genotype if dynastic effects exist.

### Determining the presence of population phenomena

Studies with a genetic focus that examine complex social phenomena such as education may be particularly susceptible to bias arising due to population-level phenomena. Education is one of many heavily studied social phenotypes in genetic studies (*24*) and is a strong determinant of health and social outcomes throughout the life course (*25*, *26*). Conceptually viewed as subcategories of broader SEP (*26*), education and occupational position are strongly correlated phenotypically and genotypically (*27*–*29*), are highly heritable (*28*–*30*), and have a complex genetic architecture characterized by high polygenicity (*24*). The heritability of education has been estimated at 40% for years of education (*30*) and 60% for test score achievement (*31*–*34*). Given this distinction, we hereafter refer to years of education as “attainment” and test score achievement as “achievement” (*35*). There is evidence of high genetic correlation (0.48 to 1) between educational attainment and other indicators of SEP such as social class (*28*, *29*), but these may operate through an intermediate phenotype such as cognitive ability (*28*). Cognitive ability is highly heritable (*29*, *31*) and correlates with many measures of SEP phenotypically and genotypically (*27*, *31*, *36*). The way in which complex social phenotypes such as education and occupation associate with genotype may have important implications for social policy to reduce inequalities throughout the life course. It is therefore of paramount importance that results from studies investigating these phenotypes are interpreted correctly with an awareness of the mechanisms by which genotype-phenotype associations can arise.

Statistical methods to estimate genetic associations from unrelated individuals often assume no unmeasured population stratification, dynastic effects, or assortative mating. Where these structures exist and are insufficiently controlled for, estimates of genetic associations will be biased due to hidden correlations in the data and incorrectly attributed to genetic effects (*37*). To empirically explore the mechanisms described above, we performed a set of analyses using the example of educational achievement, SEP, and cognitive ability in a U.K. birth cohort, the Avon Longitudinal Study of Parents and Children (ALSPAC). To demonstrate that results are not driven by genotyping errors or other biases, we also present results for C-reactive protein (CRP), a biomarker of inflammation that is associated with a range of complex diseases, as a negative control analysis (*38*). CRP is a biomarker of inflammation that is associated with a range of complex diseases, is rarely observed in younger people, and is unlikely to be influenced by assortative mating, dynastic effects, or population stratification. Systematic population differences (population stratification) in CRP have been found to be insubstantial (*39*); parental phenotypic effects of CRP are unlikely to influence offspring CRP (dynastic effects); and parents are very unlikely to selectively mate based on CRP (assortative mating). First, we present univariate heritability and genetic correlation estimates for our phenotypes. Second, we use bivariate heritability as a measure of genetic influence on phenotypic similarity between phenotypes and estimate this for each phenotype pair. Last, we present results from a range of analyses designed to assess the presence of bias due to population stratification, dynastic effects, and assortative mating.

## RESULTS

### Whole-genome estimates of genotype-phenotype associations for socioeconomic traits

#### Univariate phenotypic heritability

**Fig. 2:**
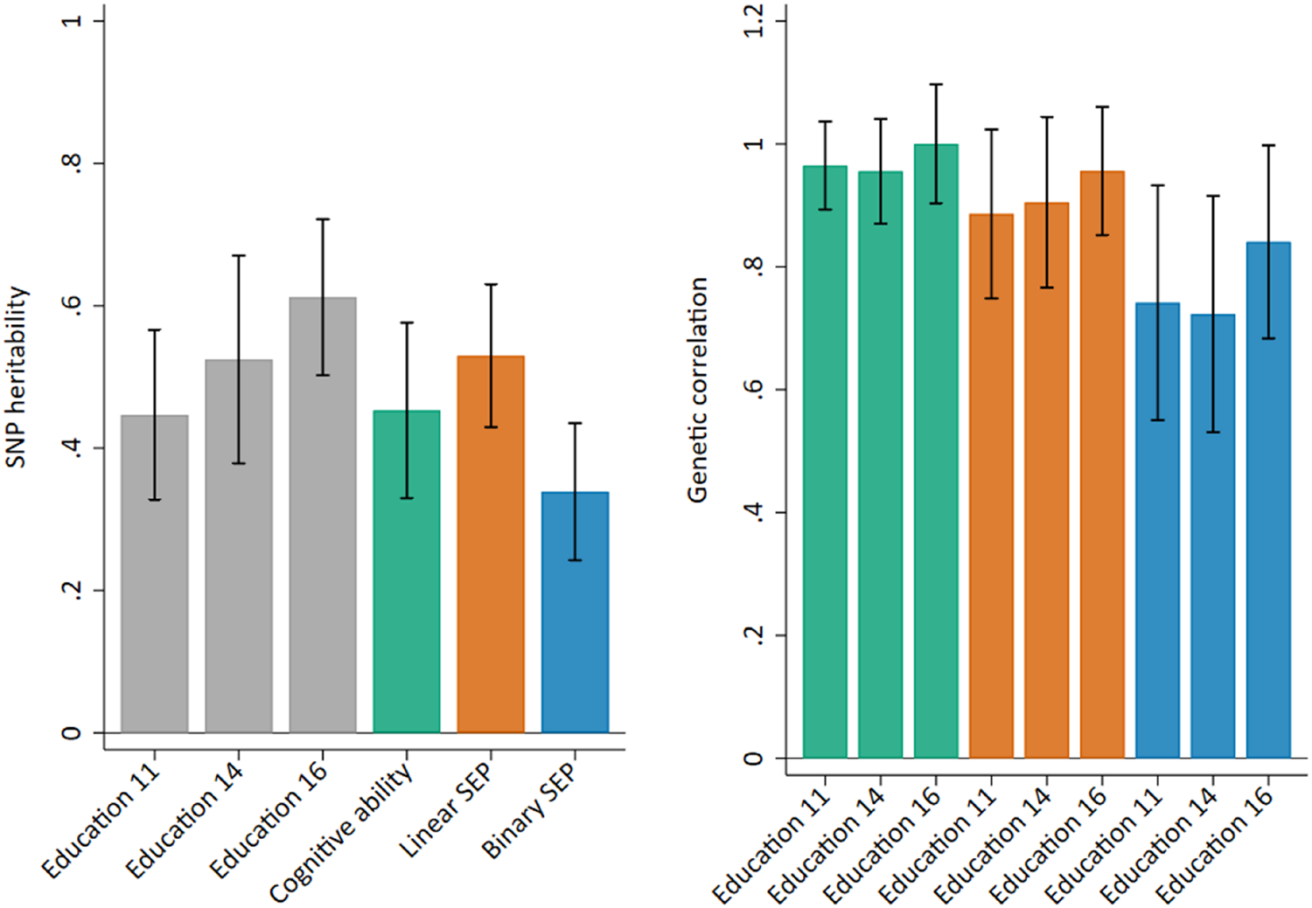
SNP heritability and genetic correlations between phenotypes. (**A**) Gray bars represent educational achievement measured as exam point scores at ages 11, 14, and 16; green bar represents cognitive ability measured at age 8; orange bar represents a linear measure of SEP measured as highest parental score on the Cambridge Social Stratification Score; blue bar represents a binary measure of SEP measured as “advantaged” for the highest two categories of Social Class based on Occupation and “disadvantaged” for the lower four categories. (**B**) Green bars represent genetic correlations between educational achievement at ages 11, 14, and 16 with cognitive ability measured at age 8; orange bars represent genetic correlations between educational achievement at ages 11, 14, and 16 with linear SEP; blue bars represent genetic correlations between educational achievement at ages 11, 14, and 16 with binary SEP. All analyses include adjustment for the first 20 principal components of population stratification. Parameter estimates in tables S1 and S2.

To investigate whether and how genotype-phenotype associations may be biased, we began by inferring the total contribution of all SNPs to the phenotypic variance, assuming an infinitesimal model of genetic architecture (*11*). The SNP heritability of educational achievement increased with age from 44.7% [95% confidence interval (CI), 32.7 to 56.6] at age 11 to 52.5% (95% CI, 37.8 to 67.0) at age 14 and 61.2% (95% CI, 50.2 to 72.2) at age 16 (Fig. 2A). The heritability of cognitive ability was estimated at 45.2% (95% CI, 33.0 to 57.6), and the heritability of SEP was estimated to be higher for a linear measure (53.0%; 95% CI, 42.9 to 63.0) than a binary measure (33.9%; 95% CI, 24.2 to 43.5).

#### Genetic correlation

We next estimated genetic correlations between each phenotype pair to infer the extent to which genetic effects were shared across phenotypes. Genetic correlations between educational achievement and cognitive ability were high and persisted throughout childhood within the range of 0.96 to 1 (Fig. 2B). This suggests that most of the SNPs that associate with educational achievement also associate with cognitive ability. Genetic correlations between educational achievement and SEP were also high: For the linear measure, they ranged from 0.89 (95% CI, 0.75 to 1.02) to 0.96 (95% CI, 0.85 to 1.06), and for the binary measure, they ranged from 0.76 (95% CI, 0.57 to 0.95) to 0.87 (95% CI, 0.71 to 1.04). The genetic correlations suggest that many SNPs that associate with educational achievement also associate with family SEP. These results were not driven by genotyping or imputation method (tables S1 to S4).

**Fig. 3:**
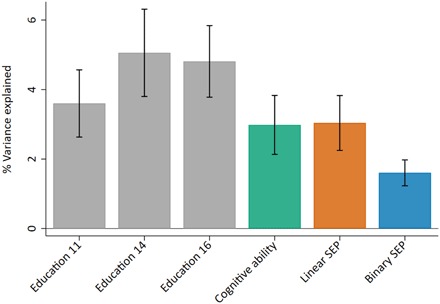
Variance explained in phenotypes by the educational achievement polygenic score. Polygenic score constructed from SNPs associated with education at *P* < 5 × 10^−8^. Gray bars represent educational achievement measured as exam point scores at ages 11, 14, and 16; green bar represents cognitive ability measured at age 8; orange bar represents a linear measure of family SEP measured as highest parental score on the Cambridge Social Stratification Score; blue bar represents a binary measure of family SEP measured as advantaged for the highest two categories of Social Class based on Occupation and disadvantaged for the lower four categories. SEs were obtained through bootstrapping with 1000 replications. All analyses include adjustment for the first 20 principal components of population stratification. Parameter estimates in table S5.

As a sensitivity analysis, we estimated the amount of variance in each phenotype that could be explained by a polygenic score for educational achievement built from the largest GWAS of educational attainment to date (using summary stats excluding the ALSPAC sample) (*24*). This explained between 3.6 and 5.1% of the variation in educational achievement, 3.0% in cognitive ability and the linear measure of SEP, and 1.6% in the binary measure of SEP (Fig. 3). That the polygenic score explains a similar amount of variation in the linear measure of SEP as educational achievement suggests a modest amount of pleiotropy in the SNPs used in the score, underscoring the high genetic correlations.

#### Bivariate SNP heritability

**Fig. 4:**
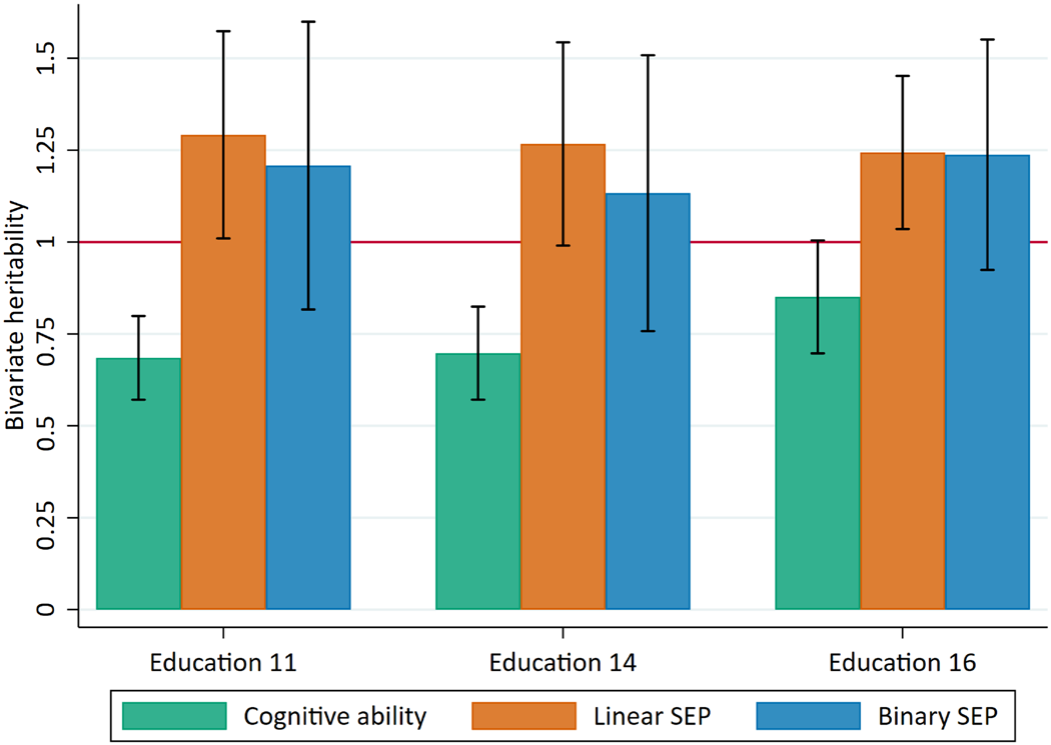
Bivariate heritabilities between educational achievement, cognitive ability, and SEP. Green bars represent cognitive ability measured at age 8; orange bars represent a linear measure of family SEP measured as highest parental score on the Cambridge Social Stratification Score; blue bars represent a binary measure of family SEP measured as advantaged for the highest two categories of Social Class based on Occupation and disadvantaged for the lower four categories. Educational achievement was measured as exam point scores at ages 11, 14, and 16. SEs were obtained through simulations (see Data and Methods). All analyses include adjustment for the first 20 principal components of population stratification. Parameter estimates in table S6.

While genetic correlation estimates the correlation between the effects of SNPs on two phenotypes, it provides no information of how important genotype effects for one phenotype are for phenotypic differences in another. Bivariate heritability, which estimates the proportion of phenotypic correlation between two traits that can be attributed to genotype (calculated as $h_{\mathrm{AB}}^{2}=\frac{r_{g}\sqrt{h_{A}^{2}}\sqrt{h_{B}^{2}}}{r_{p}}$), can be used to infer this. The bivariate heritabilities of educational achievement and cognitive ability range from 0.69 [standard error (SE), 0.06] at age 11 to 0.85 (SE, 0.08) at age 16 (Fig. 4 and table S6). At face value, this suggests that over two-thirds of the phenotypic similarity between educational achievement and cognitive ability can be explained by shared common genetic variation in our sample. The bivariate heritabilities for educational achievement and SEP were estimated at greater than one for both the linear and binary measures (Fig. 4 and table S6), and the SEs suggest that this is not solely due to estimation imprecision. Bivariate heritability estimates greater than one are mathematically plausible because they are a ratio of two terms in which the numerator is not completely nested within the denominator. It is possible that bivariate heritability estimates above one may be an unbiased reflection of negative confounding caused by an environmental factor, but this would require strong effects (see the Supplementary Material). Bivariate heritabilities greater than one can therefore be taken as an indicator that estimates of univariate heritabilities or genetic correlation may have been biased, leading to overestimation of the genetic parameter $\left(r_{g}\sqrt{h_{A}^{2}}\sqrt{h_{B}^{2}}\right)$. This information would not be obtained from genetic correlation estimates, demonstrating the usefulness of bivariate heritability for identifying the presence of bias due to population phenomena. We now investigate how these population phenomena may have biased our estimates.

### Exploring potential mechanisms of estimate inflation

#### Population stratification

Comparison of heritability estimates between models that omit and include the first 20 principal components indicates that bias due to population stratification as measured by the principal components is likely to be low (Table 1). The SEs are relatively large, and there is little evidence of differences in the heritability point estimates after additionally adjusting for the principal components. It is important to note that while adjustment for the first 20 principal components is unlikely to have removed all population stratification bias (*16*), these results suggest that bias due to population stratification is likely to be low.

**Table 1:** SNP heritability estimates of phenotypes before and after adjusting for the first 20 principal components of ancestry. SEs in parentheses. EA, educational achievement.

	**Unadjusted**	**Population** **stratification** **adjusted**
EA age 11	0.498 (0.056)	0.451 (0.064)
EA age 14	0.545 (0.069)	0.509 (0.079)
EA age 16	0.607 (0.052)	0.605 (0.059)
Cognitive ability	0.453 (0.058)	0.417 (0.066)
Linear SEP	0.568 (0.047)	0.547 (0.054)
Binary SEP	0.372 (0.046)	0.337 (0.052)

#### Dynastic effects

Table 2 shows associations between offspring education polygenic scores and educational achievement at age 16 before and after adjustment for parental polygenic scores, based on a sample of 1095 mother-father-offspring trios. In the unadjusted model, a one SD higher educational achievement polygenic score built from all SNPs is associated with a 0.340 (SE, 0.028) SD higher achievement at age 16. After adjustment for parental polygenic scores, this is attenuated to 0.223 (SE, 0.041), an attenuation of 34.4%. Using polygenic scores built only from SNPs that reached genome-wide significance, the association of polygenic scores and educational achievement attenuated by 60.5% after adjustment for parental polygenic scores. Furthermore, parental genome-wide education polygenic scores remained associated with their child’s education achievement conditional on the child’s polygenic score, suggesting the presence of dynastic effects or assortative mating. Our negative control analyses of CRP based on 942 mother-father-offspring trios showed that a one SD higher CRP polygenic score was associated with a 0.219 (SE, 0.030) SD higher level of CRP. After adjustment for parental CRP polygenic scores, this is attenuated to 0.192 (SE, 0.043), an attenuation of 12.4%. Neither the maternal nor paternal CRP polygenic scores were associated with offspring phenotypic CRP conditional on offspring CRP polygenic score, consistent with no dynastic effects for CRP as would be expected for such a biological phenotype.

**Table 2:** Associations between child and parent polygenic scores with phenotypes (education, *n* = 1095 trios; CRP, *n* = 942 trios). SEs in parentheses. Independent associations represent regression models with only a single parent polygenic score (PGS) variable included; adjusted associations represent regression models with all three PGS variables. All models control for the first 20 principal components of ancestry. *P* values obtained from tests of seemingly unrelated regression on the child PGS coefficients between independent and coadjusted models. SEs for attenuation were obtained through bootstrapping with 1000 replications.

	**Independent associations**	**Adjusted associations**	***P* value for difference**	**Attenuation in child PGS** **coefficient**
Education				
Education PGS all SNPs				
Child PGS	0.340 (0.028)	0.223 (0.041)	1.9 × 10^-4^	34.4% (9.3)
Mother PGS	0.261 (0.029)	0.110 (0.036)		
Father PGS	0.232 (0.030)	0.099 (0.034)		
Education PGS GWAS SNPs				
Child PGS	0.129 (0.029)	0.051 (0.042)	0.009	60.5% (35.0)
Mother PGS	0.072 (0.030)	0.035 (0.036)		
Father PGS	0.142 (0.029)	0.111 (0.036)		
CRP				
CRP GWAS SNPs				
Child PGS	0.219 (0.033)	0.192 (0.043)	0.330	12.4% (13.3)
Mother PGS	0.079 (0.033)	−0.002 (0.037)		
Father PGS	0.144 (0.032)	0.056 (0.037)		

#### Assortative mating

Table 3 demonstrates phenotypic and genotypic correlations for all available parental spouse pairs in the ALSPAC cohort. Phenotypic spousal correlations were positive for all phenotypes and similar to those estimated in other studies [cf 0.41 (*9*), 0.62 (*40*), and 0.66 (*41*)]. This provides evidence of phenotypic assortative mating on both education and SEP between ALSPAC parents. To test whether this phenotypic sorting induced genetic correlations between spouses, we examined genetic correlations between spouses based on education polygenic scores. Positive correlations were observed between spouse pairs for both polygenic scores, suggesting that the observed phenotypic assortment induced genetic assortment and that assortative mating likely contributed to bias in heritability estimates of educational achievement among offspring (*8*). Turning to the negative control analysis, the spousal phenotypic correlation for CRP was 0.004 (0.030), and the spousal correlation of the CRP polygenic score was −0.009 (0.027). These results contrast to the spousal correlations on the social variables and imply no assortment on CRP.

**Table 3:** Phenotypic and genotypic correlations between spouses. SEs in parentheses.

	**Spouses**	***n***
Phenotype		
Highest educational achievement	0.560 (0.011)	5353
Linear SEP	0.434 (0.010)	6858
Binary SEP	0.297 (0.008)	5737
CRP	0.004 (0.030)	1129
Genotype		
Education genetic score all SNPs	0.181 (0.025)	1262
Education genetic score GWAS SNPs	0.080 (0.027)	1262
CRP genetic score GWAS SNPs	−0.009 (0.027)	1385

**Fig. 5:**
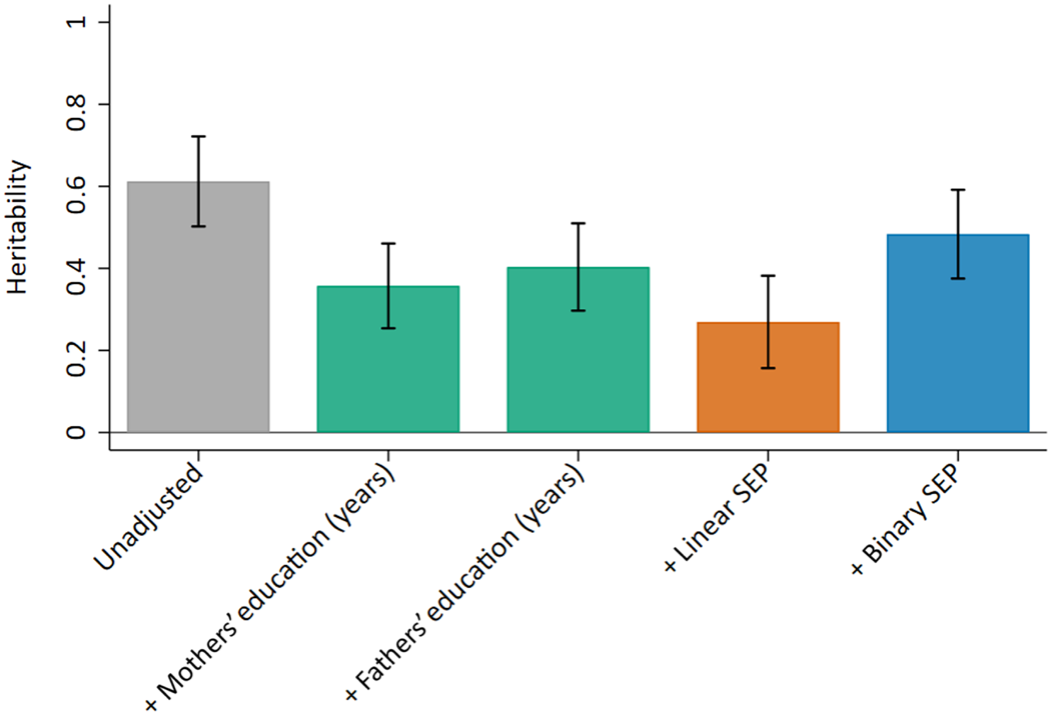
Heritability of educational achievement adjusting for parental socioeconomic variables. Gray bar represents the estimated heritability of educational achievement measured at age 16; green bars represent heritability adjusted for mothers’ and fathers’ years of education; orange bar represents heritability adjusted for linear SEP; blue bar represents heritability adjusted for binary SEP.

To further explore the potential impact of assortative mating and dynastic effects on our results, we conducted additional sensitivity analyses controlling for parents’ years of education and SEP. This approach still assumes no assortative mating or dynastic effects, but inconsistency between the main results and these sensitivity analyses provides an indication of bias in heritability due to these processes. The results of these analyses (Fig. 5) demonstrate that the heritability of educational achievement at age 16 is greatly attenuated—by around half—when parental education or SEP is controlled for. This suggests that differences in educational achievement, which are associated with common genetic variation, can, in part, be explained by assortative mating, dynastic effects, or a combination of both. When these sensitivity analyses were applied to genetic correlation estimates between education and SEP, the impact of these biasing mechanisms was less clear, reflecting greater estimation imprecision (fig. S1).

## DISCUSSION

By analyzing genetic contributions to socioeconomic phenotypes alongside a wide set of sensitivity analyses, we have demonstrated how population phenomena can bias estimates of genetic contributions to complex social phenotypes from samples of unrelated individuals. The presence of genetic association does not necessarily imply a variant substitution effect, solely giving rise to genotype-phenotype associations, but may reflect confounding by underlying population phenomena including population stratification, assortative mating, and dynastic effects. These results demonstrate that analyses using samples of unrelated individuals may not provide estimates of heritability or genetic correlation that are driven solely by causal genotype-phenotype relationships, and this likely reflects mechanisms influencing GWAS also. Our results add to the growing body of evidence that estimates drawn from samples of unrelated individuals may overestimate heritability or genetic correlation (*7*, *8*, *10*) and bias Mendelian randomization studies (*17*). Social phenotypes such as education and SEP, which are complex, highly assortative, and dynastic, appear to be particularly susceptible to bias from population phenomena. It is therefore important that studies within the rapidly growing area of sociogenomic research (*42*) test for these phenomena using the methods that we highlight and, where possible, draw upon data from family-based studies. Estimating the attenuation of offspring polygenic scores from parental polygenic scores can help to identify dynastic effects; spousal correlations can provide information on the presence of assortative mating; and bivariate heritability can be used to identify overestimation in genetic parameters as a result of these phenomena.

Our SNP heritability estimates of educational achievement were higher than those previously estimated from a different U.K. cohort at around 25% at age 7 to 40% at age 16, although the CIs between the estimates from the two studies overlap (*43*). Differences in heritability estimates cannot be taken as evidence of misestimation, though, as they are relevant to a specific population at a specific time (*44*). These SNP heritability estimates are higher than those for educational attainment (*24*, *45*), which may reflect differences in the heritability of attainment and achievement. SNP heritabilities of attainment and achievement have not yet been estimated in the same sample, but comparing different samples, achievement at the end of schooling has been estimated higher (SNP heritability, 0.4) (*43*) than lifetime attainment (SNP heritability, 0.2) (*24*). This discrepancy may reflect sample differences, and future research is required in samples with both attainment and achievement measured. Previous studies have highlighted that SNP heritability estimates are biased by family effects (*7*, *10*), and these issues may have inflated our estimates. However, the strength of bias may be smaller for education test scores (achievement) that are likely to capture a more cognitive aspect of educational performance than the more social aspect of education that years of education (attainment) capture. As has been discussed previously (*46*), the high heritabilities that we observed may also reflect genuine differences due to the spatiotemporal homogeneity of the ALSPAC cohort. The mechanisms that we investigated may also have larger effects in the ALSPAC study as a regional cohort than in other data samples; the impact of these mechanisms on more geographically dispersed studies such as UK Biobank is currently unknown.

Our estimates of the SNP heritability of cognitive ability (41.7%) and SEP (linear, 54.7%; binary, 33.7%) were broadly similar to educational achievement and also exceeded those in previous studies of 29% for cognitive ability (*29*) and 20% for SEP (*28*, *29*). That heritability was higher in the linear measure than the binary measure of SEP may reflect our cut point in determining “high” versus “low” for the binary classification or genuine differences between the two measures. The estimates of proportion of variation in all phenotypes explained by the educational attainment polygenic score were broadly consistent with previous research (*35*). Estimated genetic correlations between educational achievement, SEP, and cognitive ability were consistent with findings from other cohorts (*28*, *29*) but with greater statistical precision due to larger sample sizes and the precision of GCTA over other methods (*47*). Further research is required to investigate how these genetic associations persist into further and higher education.

Attenuation of genetic associations between children’s polygenic score and educational achievement was between one-third and two-thirds after controlling for both parents’ polygenic scores, supporting the presence of dynastic effects whereby parental genotype indirectly affects offspring phenotype. Furthermore, both parents’ scores remained robust predictors of children’s achievement over and above the child’s polygenic score. Phenotypic spousal correlations demonstrated strong evidence of parental assortative mating on educational attainment (*r* = 0.56) and SEP (*r* = 0.43), which induced genetic correlations at education-associated loci of *r* = 0.18. Heritability estimates of educational achievement were attenuated by roughly half when parental education or SEP was controlled for. This supports bias in heritability estimates due to assortative mating and/or dynastic effects in ALSPAC. We found no strong evidence that our estimates were biased by population stratification as measured by the genetic principal components, but this may reflect the inability of genetic principal components to capture subtle population structure rather than adequately control it (*12*, *16*). It is also possible that our high estimates reflected the relatively homogenous educational environment experienced by the ALSPAC cohort when compared to previous studies. Environmental homogeneity increases the proportion of variation that can be attributed to genetic effects, and the ALSPAC children were all born within 3 years and mostly experienced the same school system within the same region of the United Kingdom. Our negative control analyses provided little evidence of dynastic effects or assortative mating for CRP in our sample. While this is expected, it strengthens confidence that the dynastic effects and assortative mating that we observe for education are robust and do not arise from other issues such as genotyping errors.

Several limitations must be acknowledged in this study. First, measurement error on the phenotypes may have influenced our results. Genotyping accuracy and strict quality controls on the genetic data and educational achievement taken from administrative records should result in insufficient measurement error in these phenotypes to meaningfully bias our estimates. However, there may be some measurement inaccuracy in how well the education test scores capture underlying educational ability over and above test-retest reliability. Measurement error will be greater for SEP, as these measures relied on self-reported data, but this would have to be differential and patterned to bias estimates (independent nondifferential measurement error will only reduce statistical precision of the estimates, not bias them). Second, further residual population structure in the ALSPAC genetic relatedness matrix not captured by the principal components could bias our results (*16*). We controlled for the first 20 principal components of population structure in our full analyses, but this is unlikely to account for all differences. Another possible source of bias in our study is that of shared environmental factors (*48*) due to schooling. Many children within our sample will attend the same schools and therefore share the same schooling environment. Because school choice in the United Kingdom is socioeconomically patterned (*49*), correlations may be induced between parental SEP and school environment that would be attributed to additive genetic variation (i.e., genetic nurture effects). Recent research has demonstrated the importance of geography as a source of bias in genetic studies (*16*), and because we use a heavily geographically clustered cohort, this may bias our heritability estimates. Third, the definition of educational attainment used in the GWAS to conduct the polygenic score was years of education, which is relatively crude and does not discriminate academic performance within each additional year of education. It is therefore possible that the score we use is capturing a social rather than performance aspect of education. Fourth, GREML assumes that causal SNPs have effects on phenotypes that are independent of LD to other SNPs and minor allele frequency (MAF) (*50*). Previous studies have demonstrated that violations to these assumptions can lead to biased SNP heritability and that multicomponent GREML methods (GREML-LDMS-R and GREML-LDMS-I) can obtain accurate SNP heritability estimates (*2*, *51*). However, these extensions require much larger sample sizes to estimate than standard GREML approaches (*51*, *52*) and cannot be reliably estimated using our data. Furthermore, the attenuation that we found due to population factors is, in principle, unrelated to these potential biases that arise due to genetic architecture assumptions. Therefore, while our revised estimates may be additionally biased due to modeling assumptions, it remains likely that that would occur in addition to the population-level biases that we have described. Future studies on larger samples are required to test potential overestimation of SNP heritability for education and SEP using GREML-LDMS extensions. Last, it is possible that our estimates could have been biased by cryptic relatedness. To overcome this, we restricted our analytical sample to individuals with identity by descent (IBD) less than 0.1, but it remains possible that some related participants will have been included. While data on mother-father-offspring trios provide opportunities to investigate the presence and strength of these mechanisms, mother-father-offspring-sibling quad approaches may offer further opportunities to test for heterogeneity in dynastic effects between siblings.

In conclusion, our results demonstrate some of the causal structures that may bias univariate and bivariate genetic estimates such as heritability and genetic correlations, particularly when applied to complex social phenotypes. Future studies may make use of the methodological tools that we highlight here to assess these alongside others (*7*, *8*, *10*). Principally, family-based study designs such as within-family (*17*), between-sibling (*53*), adoption (*54*), and half-sibling (*55*) will be better equipped to provide informative and accurate genetic associations given their robustness to population stratification, dynastic effects, and assortative mating (*56*). Genetic studies investigating complex social relationships should be interpreted with care in light of these mechanisms, and results should be interpreted within a triangulation framework that considers the wider context of existing evidence (*57*).

## DATA AND METHODS

### Study sample

Participants were children from the ALSPAC. Pregnant women resident in Avon, United Kingdom with expected dates of delivery 1 April 1991 to 31 December 1992 were invited to take part in the study. The initial number of pregnancies enrolled was 14,541. When the oldest children were approximately 7 years of age, an attempt was made to bolster the initial sample with eligible cases who had failed to join the study originally. This additional recruitment resulted in a total sample of 15,454 pregnancies, resulting in 14,901 children who were alive at 1 year of age. From these, there are genetic data available for 7748 children on at least one of educational achievement, SEP, and cognitive ability after quality control and removal of related individuals (see the next section). For full details of the cohort profile and study design, see (*58*, *59*). Please note that the study website contains details of all the data that are available through a fully searchable data dictionary and variable search tool at www.bristol.ac.uk/alspac/researchers/our-data/. The ALSPAC cohort is largely representative of the U.K. population when compared with 1991 Census data; there is underrepresentation of some ethnic minorities, single parent families, and those living in rented accommodation (*58*). Ethical approval for the study was obtained from the ALSPAC Ethics and Law Committee and the Local Research Ethics Committees. Consent for biological samples has been collected in accordance with the Human Tissue Act (2004). We use the largest available samples in each of our analyses to increase precision of estimates, regardless of whether a child contributed data to the other analyses. Descriptive statistics for the raw variables used in the analyses and the phenotypic differences between ALSPAC participants included in our analyses and those who were excluded due to missing data genotype or phenotype data are in table S7. Compared to participants who were excluded due to missing data, those included in the analyses had higher achievement at each stage of education, had higher cognitive ability as measured at age 8, and came from higher SEP families as measured on both linear and binary.

### Genetic data

DNA of the ALSPAC children was extracted from blood, cell line, and mouthwash samples and then genotyped using reference panels and subjected to standard quality control approaches. ALSPAC children were genotyped using the Illumina HumanHap550 quad chip genotyping platforms by 23andMe subcontracting the Wellcome Trust Sanger Institute (Cambridge, UK) and the Laboratory Corporation of America (Burlington, NC, USA). ALSPAC mothers were genotyped using the Illumina Human660W-Quad array at Centre National de Génotypage, and genotypes were called with Illumina GenomeStudio. ALSPAC fathers and some additional mothers were genotyped using the Illumina HumanCoreExome chip genotyping platforms by the ALSPAC laboratory and called using GenomeStudio. All resulting raw genome-wide data were subjected to standard quality control methods in PLINK (v1.07). Individuals were excluded on the basis of gender mismatches, minimal or excessive heterozygosity, disproportionate levels of individual missingness (>3%), and insufficient sample replication (IBD < 0.8). Population stratification was assessed by multidimensional scaling analysis and compared with HapMap II (release 22) European descent (CEU), Han Chinese, Japanese, and Yoruba reference populations; all individuals with non-European ancestry were removed. SNPs with a MAF of <1%, a call rate of <95%, or evidence for violations of Hardy-Weinberg equilibrium (*P* < 5 × 10^–7^) were removed. Cryptic relatedness was assessed using an IBD estimate of more than 0.125, which is expected to correspond to roughly 12.5% alleles shared IBD or a relatedness at the first cousin level. Related participants that passed all other quality control thresholds were retained during subsequent phasing and imputation. For the mothers, samples were removed, where they had indeterminate X chromosome heterozygosity or extreme autosomal heterozygosity. After quality control, 9115 participants and 500,527 SNPs for the children, 9048 participants and 526,688 SNPs for the mothers, and 2201 participants and 507,586 SNPs for the fathers (and additional mothers) passed these quality control filters.

We combined 477,482 SNP genotypes in common between the samples. We removed SNPs with genotype missingness above 1% due to poor quality and removed participants with potential ID mismatches. This resulted in a dataset of 20,043 participants containing 465,740 SNPs (112 were removed during liftover and 234 were out of Hardy-Weinberg equilibrium after combination). We estimated haplotypes using ShapeIT (v2.r644), which uses relatedness during phasing. The phased haplotypes were then imputed to the Haplotype Reference Consortium (HRCr1.1, 2016) panel of approximately 31,000 phased whole genomes. The HRC panel was phased using ShapeIT v2, and the imputation was performed using the Michigan imputation server. After imputation and filtering on MAF > 0.01 and info > 0.8, there were 7,191,388 SNPs. This gave 8237 eligible children, 8675 eligible mothers, and 1722 eligible fathers with available genotype data after exclusion of related participants using cryptic relatedness measures described previously.

### Educational achievement

We use average fine graded point scores at the three major Key Stages of education in the United Kingdom at ages 11, 14, and 16. Point scores were obtained from the Key Stage 4 (age 16) database of the UK National Pupil Database (NPD) through data linkage to the ALSPAC cohort. The NPD represents the most accurate record of individual educational achievement available in the United Kingdom. The Key Stage 4 database provides a larger sample size than the earlier two Key Stage databases and contains data for each. Fine graded point scores provide a richer measure of a child’s achievement than level bandings and were therefore chosen as the most accurate method of determining academic achievement during compulsory schooling.

### Parental SEP

We use two measures of parental SEP: a binary classification based on the widely used Social Class based on Occupation (formerly Registrar General’s Social Class) of “high” (I and II) versus “low” (III-Non-manual, III-Manual, IV, and V) social classes and a continuous classification based on the Cambridge Social Stratification Score (CAMSIS). Social Class based on Occupation assumes within-strata social homogeneity with clear boundaries, while CAMSIS provides a more flexible measure that accounts for social heterogeneity.

### Cognitive ability

Cognitive ability was measured during the direct assessment at age 8 using the short-form Wechsler Intelligence Scale for Children from verbal, performance, and digit span tests (*60*) and administered by members of the ALSPAC psychology team overseen by an expert in psychometric testing. The short-form tests have high reliability, and the ALSPAC measures use subtests with reliability ranging from 0.70 to 0.96. Raw scores were recalculated to be comparable to those that would have been obtained had the full test been administered and then age-scaled to give a total overall score combined from the performance and verbal subscales.

### Educational attainment polygenic score

To test dynastic effects, we used an educational attainment polygenic score with the 1271 independent SNPs identified to associate with years of education at genome-wide levels of significance (*P* < 5 × 10^−8^) in GWAS (*24*) using the software package PRSice (*61*). PRSice was used to thin SNPs according to LD through clumping, where the SNP with the smallest *P* value in each 250-kb window was retained and all other SNPs in LD with an *r*^2^ of >0.1 were removed. This score was generated using GWAS results that had removed ALSPAC and 23andMe participants from the meta-analysis. In the GWAS, the score using the 1271 genome-wide significant SNPs explained 2.5 to 3.8% of the variation in educational attainment in the two prediction cohorts.

### Negative control analyses

To ensure that our analyses were correctly identifying the biasing mechanisms that we outline and did not represent other biasing factors such as genotyping errors, we ran sensitivity analyses using CRP as a negative control phenotype. CRP is a biomarker of inflammation that is associated with a range of complex diseases and is unlikely to be influenced by the biasing mechanisms that relate to the social phenotypes that we investigate. Offspring CRP was measured from nonfasting blood assays taken during direct assessment when the children were aged 9.

### Statistical analysis

We estimate SNP heritability (hereafter referred to as heritability) using GREML in the software package GCTA (*11*). GCTA uses measured SNP-level variation across the whole genome to estimate the proportion of variation in educational achievement, SEP, and cognitive ability that can be explained by common genetic variation. We use a series of univariate analyses of the form

y=Xβ+g+ϵ (1)

where *y* is the phenotype, *X* is a series of covariates, *g* is a normally distributed random effect with variance $\sigma_{g}^{2}$, and ϵ is residual error with variance $\sigma_{\epsilon }^{2}$. Heritability is defined as the proportion of total phenotypic variance (genetic variance plus residual variance) explained by common genetic variation

$$\frac{\sigma_{g}^{2}}{\sigma_{g}^{2}+\sigma_{\epsilon }^{2}}$$ (2)

Where genetically similar pairs are more phenotypically similar than genetically dissimilar pairs, heritability estimates will be nonzero.

We estimate genetic correlations using bivariate GCTA, running nine sets of analyses between educational achievement at each age and each of linear SEP, binary SEP, and cognitive ability. Genetic correlation quantifies the extent to which SNPs that associate with one phenotype (i.e., educational achievement) also associate with another phenotype (i.e., cognitive ability). It therefore refers to the correlation of all genetic effects across the genome for phenotypes *A* and *B* and is estimated as

$$r_{g}=\frac{{\text{cov}}_{g}(A,B)}{\sqrt{{\text{var}}_{g}(A){\text{var}}_{g}(B)}}$$ (3)

where *r*_g_ is the genetic correlation between phenotypes *A* and *B*, var_g_(*A*) is the genetic variance of phenotype *A*, and cov_g_(*A*, *B*) is the genetic covariance between phenotypes *A* and *B*. Genetic correlations can indicate that two phenotypes are influenced by the same SNPs (i.e., have shared genetic architecture). In contrast, the bivariate heritability is the proportion of the phenotypic correlation that can be explained by the genotypes. Genetic correlations and bivariate heritability are likely to differ. For example, two phenotypes may be highly genetically correlated, but if they have low heritability, then the bivariate heritability will be low. Bivariate heritability estimates the proportion of phenotypic correlations that can be explained by genetics. It is estimated as

$$h_{\mathrm{AB}}^{2}=\frac{r_{g}\sqrt{h_{A}^{2}}\sqrt{h_{B}^{2}}}{r_{p}}$$ (4)

where *r*_g_ is the genetic correlation between phenotypes *A* and *B*; $h_{A}^{2}$ and $h_{B}^{2}$ are the heritabilities of phenotype *A* and *B*, respectively; and *r*_p_ is the phenotypic correlation between phenotypes *A* and *B*. GCTA does not directly estimate coheritability or bivariate heritability terms and therefore cannot be used to estimate SEs. Bivariate heritability estimates were derived from entering the GCTA estimated components into Eq. 4 above. SEs for the bivariate heritability estimates were estimated using simulations. First, we simulated 1 million observations for each input parameter (the two heritability terms, genetic correlation and phenotypic correlation) as normally distributed with a mean value corresponding to the point estimate and SD corresponding to the SE obtained from GCTA. Bivariate heritability was estimated for each observation and the SD of all 1 million estimates taken as the estimated error for the point estimate. This approach to calculating SEs assumes no covariance between the input parameters. Nonzero covariances would produce smaller SEs, and therefore, this approach can be considered to provide conservative estimates.

We used data for unrelated participants, as indicated by the ALSPAC genetic relatedness matrices. Population stratification is controlled for by using the first 20 principal components of inferred population structure as covariates in analyses. Continuous variables were inverse normally transformed to have a normal distribution, a requirement of GCTA.

## Supplementary Material

aay0328_SM.pdf

aay0328_Data_file_S1.xlsx

## SUPPLEMENTARY MATERIALS

Supplementary material for this article is available at http://advances.sciencemag.org/cgi/content/full/6/16/eaay0328/DC1

## Appendix

View/request a protocol for this paper from *Bio-protocol*.
