# Supplementary material for: Population phenomena inflate genetic associations of complex social traits
Source: Sci Adv. 2020 Apr 15;6(16):eaay0328. doi: 10.1126/sciadv.aay0328 (PMC7159920; doi:10.1126/sciadv.aay0328)

Science Advances
AAAS

advances.sciencemag.org/cgi/content/full/6/16/eaay0328/DC1

# Supplementary Materials for

## Population phenomena inflate genetic associations of complex social traits

Tim T. Morris*, Neil M. Davies, Gibran Hemani, George Davey Smith

*Corresponding author. Email: tim.morris@bristol.ac.uk

Published 15 April 2020, *Sci. Adv.* **6**, eaay0328 (2020)
DOI: 10.1126/sciadv.aay0328

**The PDF file includes:**

Figs. S1 to S4
Negative confounding

**Other Supplementary Material for this manuscript includes the following:**

(available at advances.sciencemag.org/cgi/content/full/6/16/eaay0328/DC1)

Data file S1

**Figure S1: Genetic correlations between education and socioeconomic position.** Left panel: genetic correlations between educational attainment and linear socioeconomic position. Right panel: genetic correlations between educational attainment and binary socioeconomic position. Grey bars represent the estimated genetic correlation between educational achievement measured at age 16 and socioeconomic position; green bars represent genetic correlation adjusted for mothers and fathers' years of education.

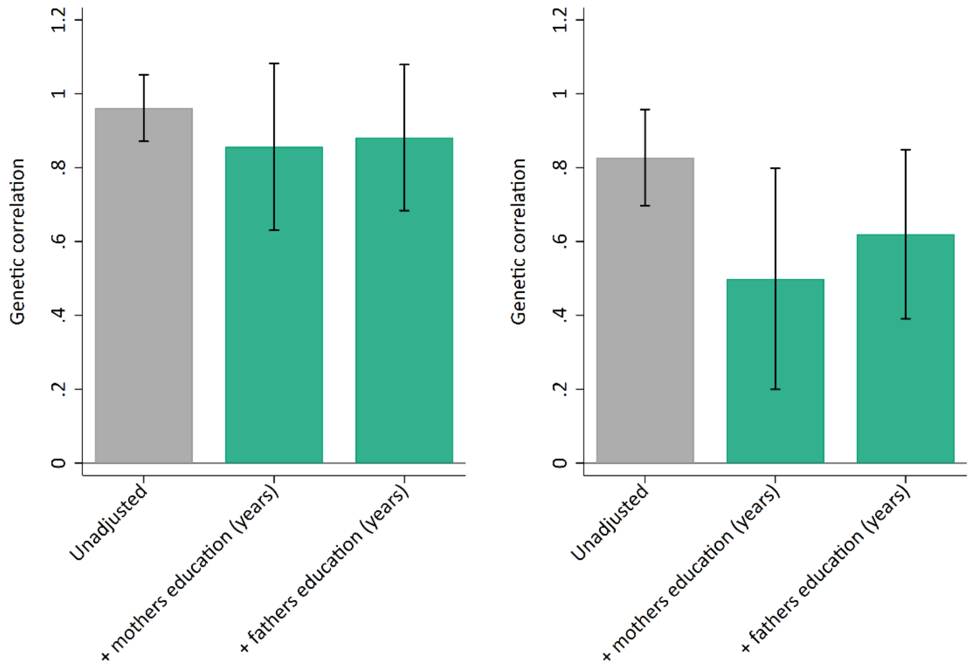

## Negative confounding

Bivariate heritabilities describe the proportion of phenotypic similarity between two traits that is due to genetic similarity. As such, bivariate heritability estimates are expected to be constrained to values within the range of zero to one, where zero indicates no genetic influence on phenotypic similarity and one indicates total genetic influence on phenotypic similarity. However, bivariate heritability is estimated using covariance terms and therefore estimates are not mathematically constrained by the upper bound of 1. One reason that bivariate heritability may be estimated at greater than one is the presence of negative confounding, whereby a secondary variable drives associations by acting in the opposite direction on two traits. Negative confounding can therefore lead to artificial reduction of phenotypic correlations between two phenotypes, increasing the proportion of estimated phenotypic similarity that can be explained by genetic similarity and leading to higher bivariate heritability estimates. Examples of negative confounding between educational achievement and socioeconomic position caused by an environmental factor in our study could include:

1. Positive discrimination in educational settings whereby children are treated differently depending on their social background;
2. Greater educational support for children from disadvantaged social backgrounds.

To investigate the amount of negative confounding that would be required to obtain bivariate heritabilities greater than one given our estimates, we ran a series of simulations. For each phenotype-pair we defined the variables according to the variation they explained in two phenotypes $A$ and $B$ ($u_A^2$ and $u_B^2$ respectively) and the correlation between these effects ($r_u$).

The model underlying the simulations was as follows:

$$y_A = g_A + u_A + e_A$$

$$y_B = g_B + u_B + e_B$$

$$g_A \sim g_B \sim MVN\left(0, \Sigma_g^2\right)$$

$$u_A \sim u_B \sim MVN(0, \Sigma_u^2)$$

$$e_A \sim N\left(0, 1 - \sigma_{gA}^2 - \sigma_{uA}^2\right)$$

$$e_B \sim N\left(0, 1 - \sigma_{gB}^2 - \sigma_{uB}^2\right)$$

$$\Sigma_g^2 = \begin{bmatrix} \sigma_{gA}^2 & \rho_g \\ \rho_g & \sigma_{gB}^2 \end{bmatrix}$$

$$\Sigma_u^2 = \begin{bmatrix} \sigma_{uA}^2 & \rho_u \\ \rho_u & \sigma_{uB}^2 \end{bmatrix}$$

Where $y_A$ is phenotype A, $y_B$ is phenotype B, $g_A$ and $g_B$ denote genetic effects for phenotypes A and B drawn from a multivariate normal distribution with variance $\Sigma_g^2$, $u_A$ and $u_B$ denote the effects of a confounder variable $u$ on phenotypes A and B drawn from a multivariate normal distribution with variance $\Sigma_u^2$, and $e_A$ and $e_B$ denote normally distributed residuals for phenotypes A and B. The confounder is specified in two ways:

1. With respect to the proportion of variance that it explains in phenotypes $y_A$ and $y_B$ ($\sigma_{uA}^2$ and $\sigma_{uB}^2$ respectively);
2. The correlation between $u_a$ and $u_b$ ($\rho_u$).

The full range of values (0:1) that $\sigma_{uA}^2$, $\sigma_{uB}^2$ and $\rho_u$ can take to satisfy a bivariate heritability estimate that is greater than one, given the observed heritabilities, genetic correlation and phenotypic correlations, can therefore be computed and plotted for each pair of traits. R code for generating these scenarios (available here).

Figures S2-S4 illustrate the range of values of $u_A^2$, $u_B^2$ and $r_u$ that were required to produce bivariate heritability estimates greater than one for each phenotype-pair. The pattern and strength of negative confounding is consistent across all ages of education and both measures of SEP. The simulations demonstrated that negative confounding was a possible driver of the high bivariate heritabilities we observed, if the confounder explained a high amount of variance in either phenotype; if there was a strong correlation between its effects on the phenotypes; or a more moderate function of both.

**Figure S2: Negative confounding required to obtain a bivariate heritability greater than one for educational achievement at age 16 and socioeconomic position given observed parameter estimates.** The surface represents the threshold where bivariate heritabilities exceed one. $\sigma_{uA}^2$, heritability of negative confounder for phenotype $A$; $\sigma_{uB}^2$, heritability of negative confounder for phenotype $B$; $r_u$, correlation between effects of confounder on phenotypes $A$ and $B$.

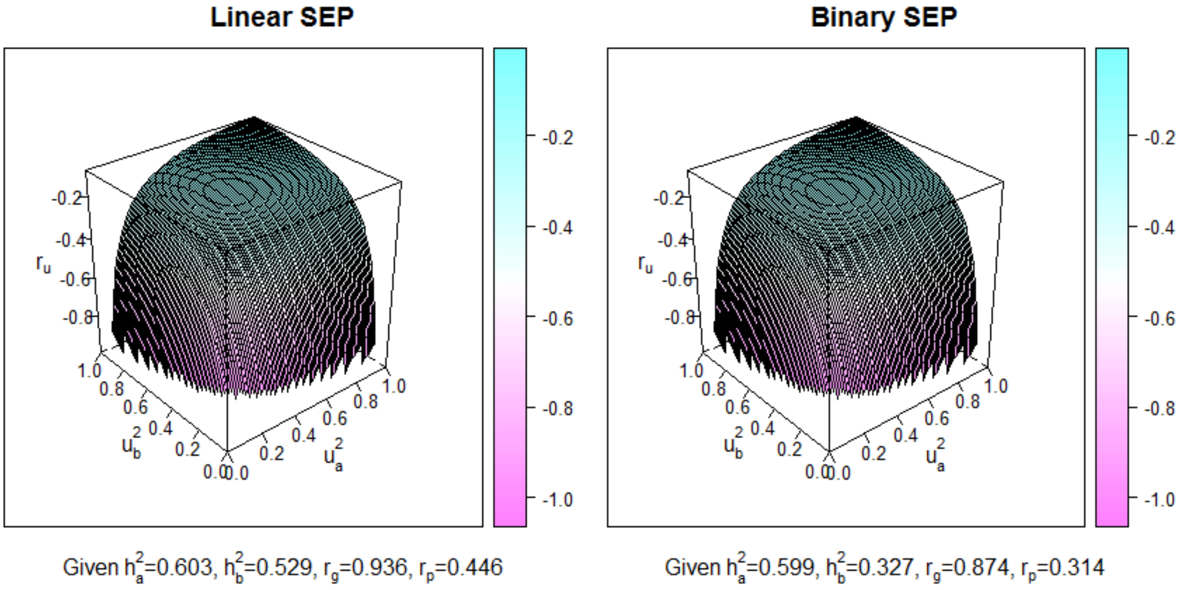

**Figure S3: Negative confounding required to obtain a bivariate heritability greater than one for educational attainment at age 11 and socioeconomic position.** The surface represents the threshold where bivariate heritabilities exceed one. $\sigma_{uA}^2$, heritability of negative confounder for phenotype $A$; $\sigma_{uB}^2$, heritability of negative confounder for phenotype $B$; $r_u$, correlation between effects of confounder on phenotypes $A$ and $B$.

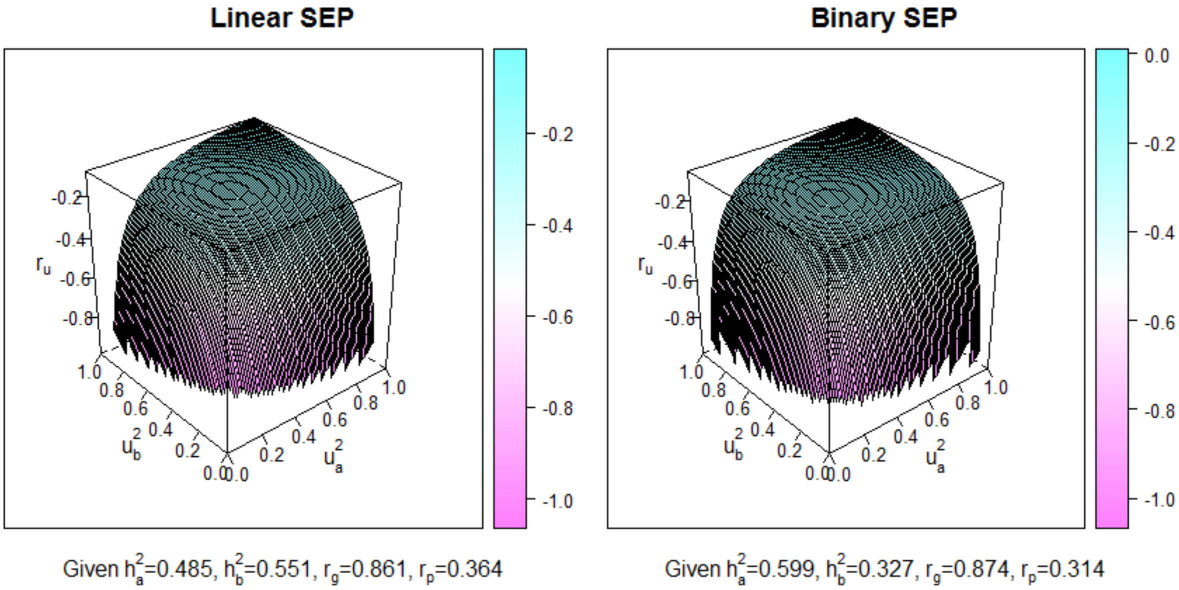


**Figure S4: Negative confounding required to obtain a bivariate heritability greater than one for educational attainment at age 14 and socioeconomic position.** The surface represents the threshold where bivariate heritabilities exceed one. $\sigma_{uA}^2$, heritability of negative confounder for phenotype $A$; $\sigma_{uB}^2$, heritability of negative confounder for phenotype $B$; $r_u$, correlation between effects of confounder on phenotypes $A$ and $B$.

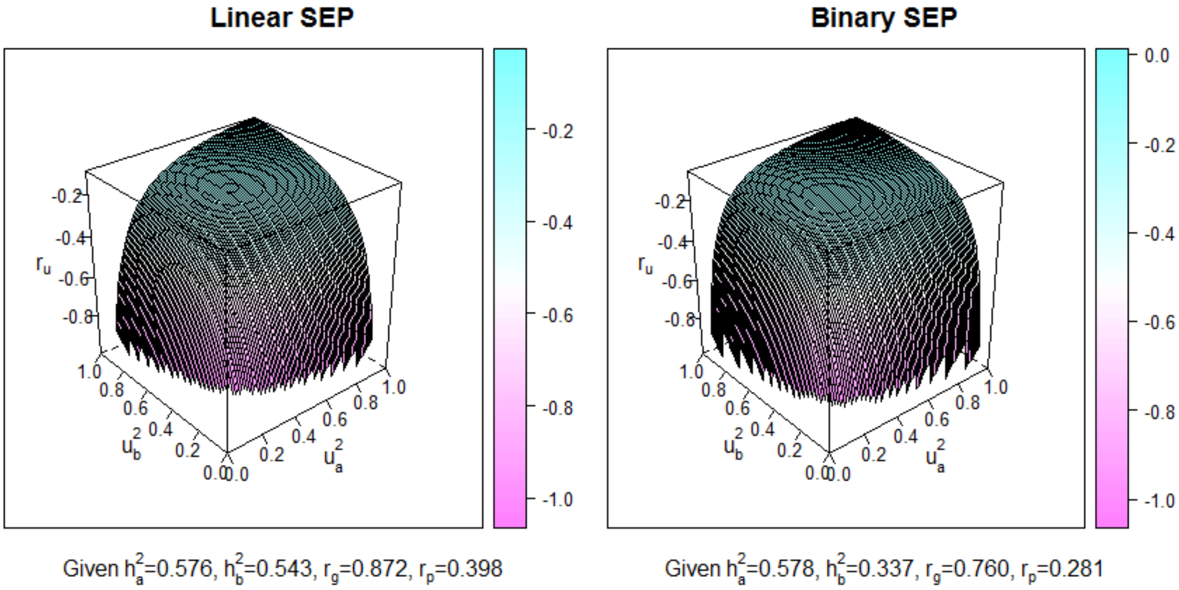

Supplement: aay0328_SM.pdf [file aay0328_SM.pdf]
